# Materials and Device Considerations in Electrophoretic Drug Delivery Devices

**DOI:** 10.1038/s41598-020-64114-0

**Published:** 2020-04-28

**Authors:** Shao-Tuan Chen, Christopher M. Proctor, George G. Malliaras

**Affiliations:** Electrical Engineering Division, Department of Engineering, University of Cambridge, Cambridge, CB3 0FA UK

**Keywords:** Biomedical engineering, Actuators

## Abstract

Electrophoretic drug delivery devices are able to deliver drugs with exceptional temporal and spatial precision. This technology has emerged as a promising platform for treating pathologies ranging from neuropathic pain to epilepsy. As the range of applications continues to expand, there is an urgent need to understand the underlying physics and estimate materials and device parameters for optimal performance. Here, computational modeling of the electrophoretic drug delivery device is carried out. Three critical performance indices, namely, the amount of drug transported, the pumping efficiency and the ON/OFF ratio are investigated as a function of initial drug concentration in the device and fixed charge concentration in the ion exchange membrane. The results provide guidelines for future materials and device design with an eye towards tailoring device performance to match disease-specific demands.

## Introduction

Neurological disorders affect over 6% of the world population and cause major economic losses. Existing treatments for these disorders are often ineffective, as systemic drug administration to the central nervous system (CNS) is impeded by the blood-brain-barrier^1^. Localized drug delivery methods such as convection-enhanced delivery devices (CED) can bypass this physiological barrier by infusing drug solution directly to the targeted area under high pressure with a catheter^2^. However, CED causes pressure increase in the targeted areas, leading to edema and disruption of adjacent neural networks^3^. Electrophoretic drug delivery devices have shown promise for a variety of eventual clinical applications in the treatment of neuropathic pain^4^ and epilepsy^5^. In these devices, drug ions are transported by electrophoresis from an internal source reservoir through an ion exchange membrane into the targeted treatment area. The device can be implanted in the targeted area, therefore bypassing the blood-brain barrier. Also, due to the ion exchange membrane, electrophoretic drug delivery devices offer the benefit of “dry” delivery as they transport only the drug without the solvent, avoiding the possibility of pressure buildup and edema in the targeted area. A key consideration for any localized drug delivery technology is the need to tailor performance to the targeted disease.

For instance, the treatment protocol for epilepsy might involve a chronic implantation for the delivery of small bursts of drugs before seizures, followed by extended periods of no drug delivery. However, for disease such as brain cancer, the treatment might involve a single dose delivered over a rather short period in an acute setting. Tailoring the performance of electrophoretic drug delivery devices to meet these varied demands however requires a deeper understanding of the device physics and trade-off between materials and device parameters. To date, our understanding of ion exchange membranes, the most critical component in an electrophoretic drug delivery device, is largely based on the research from water desalination and filtration communities^6,7^. However, there are significant differences in the operating voltage and ion concentration in the source reservoir between a typical water filtration plant and a drug delivery device. The dimensions of the source reservoir are also scaled down from meter-range for the industrialized filtration plant to micrometer-range for microfluidic-based drug delivery devices. This difference in scale accentuates physical phenomena at microscale, such as concentration polarization^8^ and capacitive charging of electrodes^9^. The above reasons necessitate further investigation of ion exchange membranes in the context of electrophoretic drug delivery devices and biological interfacing.

In this paper, we present a computational model for electrophoretic drug delivery devices based on ion transport in a typical device geometry and using appropriate initial and boundary conditions. We discuss the characteristics of these devices under different operating conditions. Three primary performance indices that describe the drug delivery process are investigated, namely, the amount of drug transported, the pumping efficiency, and the ON/OFF ratio. The results provide insight into how membrane properties and initial drug concentration affect the delivery process and therefore inform future device design and materials development.

## The model

**Figure 1:**
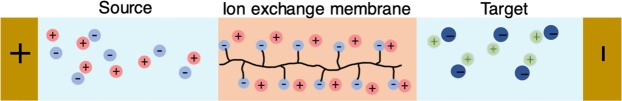
A schematic showing the components of the computational model (not to scale): source and target solutions, ion exchange membrane and capacitive electrodes.

The electrophoretic drug delivery device we investigate consists of three main components: the source reservoir loaded with drug solution, an ion exchange membrane (IEM) and a target electrolyte (Fig. 1). Metal electrodes in the source and target form the plates of a capacitor. We represent the electrophoretic drug delivery device with a one-dimensional model and solve the corresponding governing equations with finite-element based simulation software^10^. The motion of charged ions is described by the Nernst-Planck equation and combined with Poisson’s equation, which relates the charge density to the external applied electric field, forms the governing equations for the computational model of the device (see Supporting Information). The computation domain consists of source and target solutions both of which have a length *L* of 100 µm, separated by an IEM with width *L*_*m*_ of 10 µm, consistent with experimentally reported values for the dimensions of a microfluidic ion pump^4,5,11^. As for the boundary condition, the electrodes are considered to be blocking towards ions, while no additional restrictions on the flow of ions are imposed the source/IEM and IEM target interfaces. A list of variables used in this study can be found in Table S1.

At *t* = 0, the source reservoir is filled with cationic drugs and co-ions of the same initial concentration, i.e., *C*_*D*_ = *C*_*Co*_. The immobilized charge groups in the IEM for our study are negatively charged, making the IEM a cation selective membrane. For different initial drug concentration *C*_*D*_ and different fixed charge concentration *C*_*IM*_, the mobility *µ* for both the drug and co-ion in solution were set to be *µ*_*D*_ = *µ*_*Co*_ = 5 × 10^−11^ m^2^/s, consistent with previously reported values for common neurotransmitters^12^. In the IEM, all fixed charge groups are initially compensated by the cationic drug at *t* = 0, i.e., the initial drug concentration in the membrane is equal to *C*_*IM*_. The mobility of ions in the IEM is set to be 10% of their respective values compared to the mobility of these ions in solution, consistent with previously reported values^6,13^. Finally, the target is taken to be a 160 mM NaCl solution to mimic physiological conditions.

**Figure 2:**
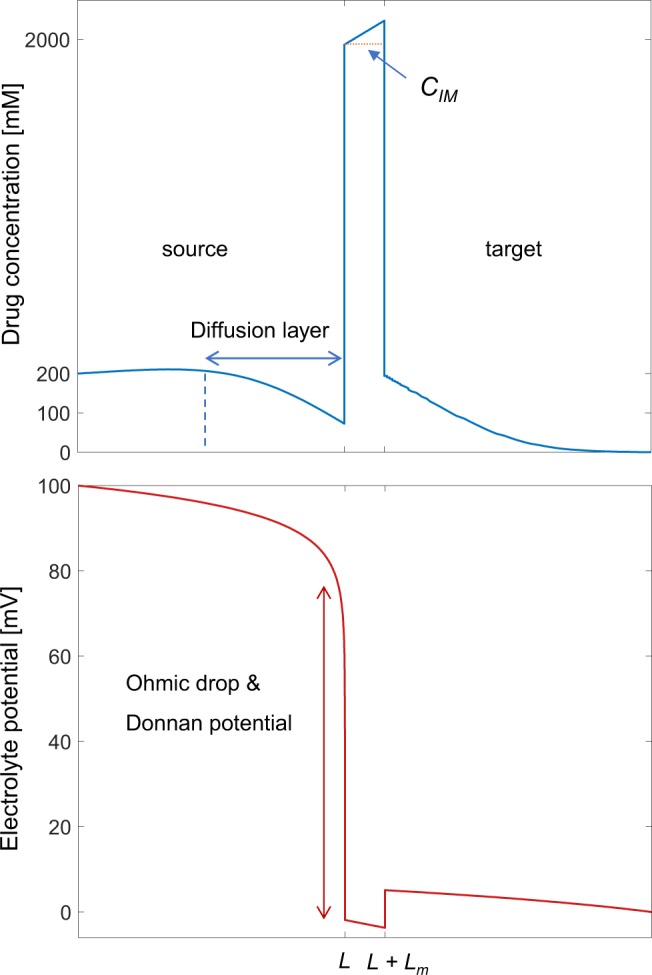
Snapshots of drug concentration (top) and voltage (bottom) profiles at *t* = 20 s. Initial drug concentration *C*_*D*_ is 200 mM, and fixed charge concentration *C*_*IM*_ is 2 M. Reservoir length *L* = 100 µm and membrane thickness *L*_*m*_ = 10 µm.

As a starting point, we employ the computational model to understand the spatial distribution of drug concentration and voltage in the device. Figure 2 shows drug concentration and voltage profiles for an applied potential of 0.5 V with *C*_*D*_ = 200 mM and *C*_*IM*_ = 2 M at 20 s. The drug concentration in the source is observed to be stable for roughly half the length of the reservoir before decreasing closer to the IEM. This concentration gradient is caused by concentration polarization^8^, as co-ions in the source reservoir are driven away from the IEM and counter ions (drug) follow the same concentration profile to maintain electroneutrality. The depletion zone describes the region adjacent to the IEM which is affected by concentration polarization. Within the depletion zone, the driving force for the drug from both applied potential and concentration gradient will drive the cationic drug towards the IEM. Concentration polarization also causes the influx of drug from the source to the IEM to be higher than the influx of drug from the IEM to the target, resulting in a drug concentration gradient opposite to the concentration gradient in the source. After the drug is delivered to the target, it moves away from the IEM, driven by both the concentration gradient and the applied potential.

The potential drop in the device shown in the bottom of Fig. 2 is from a combination of ohmic drop due to electrolyte resistance and Donnan potential at solution/IEM interfaces^14^. Between the electrodes, the potential drop is determined by ionic resistance, therefore the electrolyte potential gradually drops along the source to target direction. At the source/IEM and IEM/target interfaces, the potential drop is due to the Donnan potential^14^, which arises from ion concentration differences. The Donnan potential causes the negatively charged ions in the electrolyte to be electrostatically blocked from entering the IEM, known as the Donnan exclusion effect.

## Performance indices

For an electrophoretic drug delivery device, a critical performance index is the total amount of drug the device can deliver upon application of a voltage. One would like to control this quantity as some applications require bursts of high dosage, while other needs low and sustained delivery. The pumping efficiency η, defined as the ratio between drug transport over the total amount of ions transported, is another important quantity. Despite the selectivity of ion exchange membranes, co-ions in the source reservoir and ions in the target may still be driven by the electric field concentration gradient, thus passing through the IEM, so η is not necessarily equal to one^15^. Lastly, the ability to resist passive leakage for electrophoretic drug delivery devices is characterized by the ON/OFF ratio, which is defined as the ratio of amount of transported drug between active pumping (voltage on) and passive diffusion (voltage off). Poor control of passive diffusion can lead to chronic subtherapeutic levels of drug, which in not desirable.

The capacitive charging in the electrodes will gradually screen out the applied voltage. Therefore, the response time for capacitive charging will determine the active pumping time for an electrophoretic drug delivery device. The amount of drug transported (*Q)* by an electrophoretic drug delivery device was obtained by integrating the influx to obtain *Q* as a function of time. To avoid numerical noise caused by discontinuities at the interface between the IEM and target^16^, we performed numerical integration on influx of drug transported to target at a short distance (20 µm) away from the IEM/target interface to calculate *Q*.

**Figure 3:**
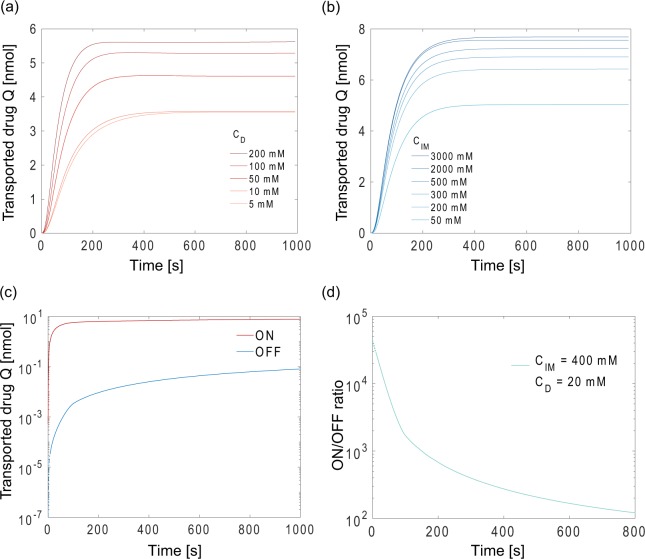
Transported drug *Q* as a function of time with (**a**) various initial drug concentration *C*_*D*_ where *C*_*IM*_ = 2 M, and (**b**) various fixed charge concentration *C*_*IM*_ in the IEM, where *C*_*D*_ = 50 mM. (**c**) Comparison between amount of drug delivered by active pumping and passive diffusion with *C*_*D*_ = 20 mM and *C*_*IM*_ = 400 mM. Due to numerical noise in the simulation, the first 5 seconds of the passive leakage data are extrapolated with exponential regression. (**d**) Resulting transient ON/OFF ratio from (**c**) as a function of time for an electrophoretic drug delivery device.

The temporal response of an electrophoretic drug delivery device is shown in Fig. 3(a). The voltage (0.5 V) is applied at t = 0, and the electric field is gradually screened out by capacitive charging leading to a saturation in the net transported drug after a few hundred seconds. The total amount of drug transported at steady-state (defined as *Q* at t = 1000 s) increases with *C*_*D*_, as the concentration gradient at the source reservoir is steeper for higher *C*_*D*_. Before the electrodes are fully charged, the instantaneous drug delivery, represented by the slopes of the curve in Fig. 3(a), is also higher with higher *C*_*D*._ Figure 3(b) shows the amount of drug delivered *Q* as a function of time for different *C*_*IM*_ with *C*_*D*_ = 50 mM. When the fixed charge concentration *C*_*IM*_ in the membrane is increased, the number of sites in the IEM available to attract the drug increases (i.e. increased ion exchange capacity^17^) leading to an increase in the flux of drug from source to target. This suggests that developing IEM materials with a higher *C*_*IM*_ could be a viable path forward to increasing drug delivery capacity.

In previous studies, the ON/OFF ratio for devices has been reported as a single value at discrete timepoints, where ON/OFF ratio is acquired experimentally by comparing the transported drug with and without an applied voltage for a given duration^11,18–20^, or theoretically by performing steady-state calculations with a numerical model^12^. However, the transient response of the ON/OFF ratio in a device with capacitive electrodes has not been explored. By calculating the amount of drug transported between active pumping and passive diffusion, we can acquire the transient ON/OFF ratio with our computational model.

For active pumping, the maximum flux of drug occurs at the start upon applying a voltage and gradually decreases thereafter, due to capacitive charging of the electrodes. In contrast, in the OFF state, the driving force is the steep concentration gradient at the IEM/target interface. Since no potential is applied during the OFF state, the concentration in the IEM remains nearly constant during passive diffusion, and the variation of concentration gradient is negligible at the IEM/target interface. As a result, the passive diffusion process when the device is switched off can be treated as ion diffusion with constant permeability through an ion exchange membrane. Figure 3(c) demonstrates the amount of transported drug *Q* between when the device is switched ON and OFF, and Fig. 3(d) shows the corresponding transient ON/OFF ratio of an electrophoretic drug delivery device. The initial concentrations of *C*_*D*_ and *C*_*IM*_ for Fig. 3(c,d) are similar to previously reported values when delivering GABA for seizure control^11^ with *C*_*D*_ = 20 mM and *C*_*IM*_ = 400 mM. From 0–100 s, the ON/OFF ratio starts at around 40000 and quickly decreases as the capacitive charging of the electrodes screen out the applied potential. After the applied potential is fully screened out, passive diffusion dominates for both ON and OFF scenarios, and the ON/OFF ratio finally decays to around 120 at *t* = 800 s.

**Figure 4:**
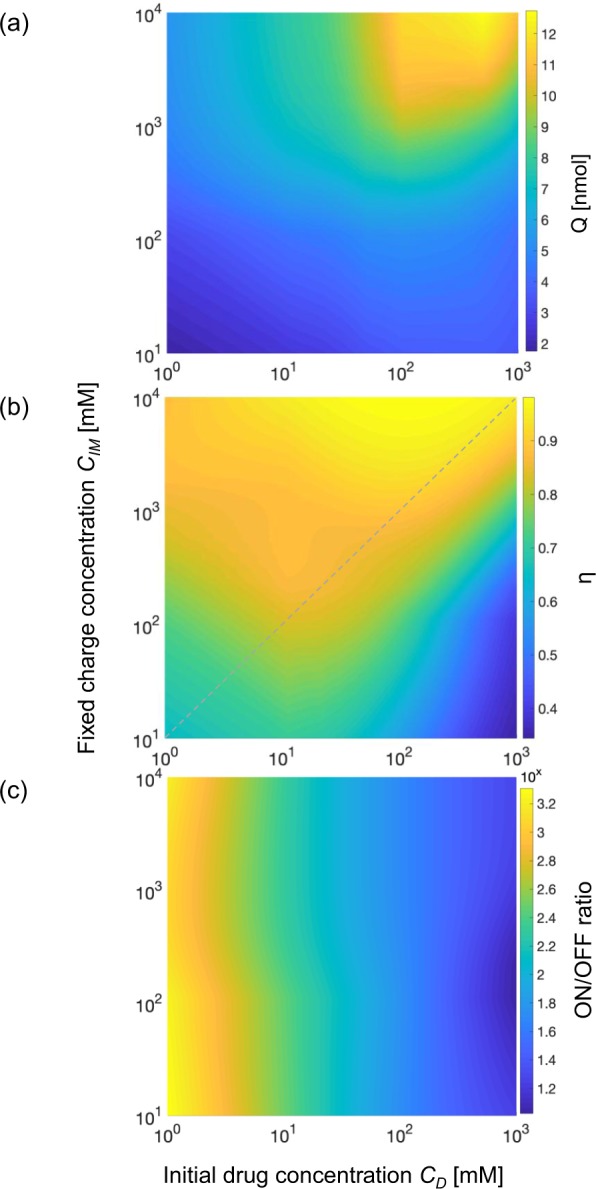
Contour plot of: (**a**) amount of drug transported at steady-state (**b**) pumping efficiency *η*, where the dotted line represents the concentration ratio for stable pumping efficiency and (**c**) ON/OFF ratio as function of both *C*_*D*_ and *C*_*IM*_, both varying by three orders of magnitude. For all contour plots, the x-axis is the initial drug concentration and y-axis is the fixed charge concentration in the IEM.

The above results demonstrate that capacitive charging of the electrodes dominates the temporal characteristics of electrophoretic drug delivery devices. However, the outcome at the end of the capacitive charging of the electrodes (steady-state) is also of interest, as it can inform applicability to different treatment scenarios. The steady-state performance index contour plots are shown in Fig. 4. Both the initial drug concentration *C*_*D*_ and the fixed charge concentration in the IEM *C*_*IM*_ were varied by three orders of magnitude (with *C*_*D*_ ranging from 1 mM to 1 M, and *C*_*IM*_ ranging from 10 mM to 1 M), a parameter space that is larger than in previous studies^5,21,22^. The contour plot for *Q* in Fig. 4(a) shows that the maximum is obtained for the highest values of initial drug concentration *C*_*D*_ and fixed charge concentration *C*_*IM*_ in the IEM. Increasing these concentrations reduces the ionic resistance of the electrolyte. For weak electrolytes, the ionic resistance can be approximated as inversely proportional to square root of electrolyte concentration^23^. As a result, increasing the concentration *C*_*D*_ or *C*_*IM*_ by 2 orders of magnitude should increase the amount of transported drug *Q* by 10-fold. However, reducing the resistance for the target and ion exchange membrane would also leads to a shorter pumping time for the device before the capacitive electrodes are fully charged^24^. As a result, increasing *C*_*D*_ and fixed *C*_*IM*_ would increase the initial drug transport but reduces the active pumping time for an electrophoretic drug delivery device. Therefore, as depicted in Fig. 4(a), by increasing *C*_*D*_ and *C*_*IM*_ for a span of 3 orders of magnitude, the amount of transported drug at steady-state can only be increased by 6-fold.

Figure 4(b) shows the contour plot for pumping efficiency *η* as a function of *C*_*D*_ and *C*_*IM*_. For different initial drug concentration *C*_*D*_, *η* increases with *C*_*IM*_. When the concentration ratio between *C*_*IM*_ to *C*_*D*_ increases, the Donnan potential between the electrolyte and IEM increases. As a result, the increased repulsive force between co-ions and the fixed charged ions in the IEM leads to higher degree of exclusion of co-ions (Donnan exclusion), and also a higher pumping efficiency *η* for the electrophoretic drug delivery device. Previous research into IEMs has shown that membrane selectivity generally exceeds 0.9 when the fixed ion concentration is at least ten times the electrolyte concentration^25^. The simulation results here indicate that a similar trend holds true in terms of pumping efficiency *η* for electrophoretic drug delivery devices in the case that *C*_*D*_ is at least 10 mM (see dashed line). However, Fig. 4(b) also shows this rule of thumb starts to break down at lower source concentrations (*C*_*D*_ < 10 mM), with nearly 100-fold greater *C*_*IM*_ required to approach pumping efficiencies >0.9. This can be understood by considering the comparatively high concentration of Cl^−^ on the target side (160 mM) is not adequately blocked by the IEM for *C*_*IM*_ < 100 mM, and therefore an increasingly significant portion of the ionic current across the IEM is attributable to Cl^−^ flowing from target to source when the device is during operation.

The ON/OFF ratio contour plot of the device at steady-state is shown in Fig. 4(c). We found that the ON/OFF ratio is very sensitive to changes in initial drug concentration *C*_*D*_, as the ON/OFF ratio at steady-state is higher than 1000 when *C*_*D*_ is lower than 5 mM. For *C*_*D*_ between 10-100 mM, the ON/OFF ratio is around 10^2^, and the ON/OFF ratio decreases to around 10 when we increase *C*_*D*_ to 1 M. The ON/OFF ratio at steady-state (t = 1000 s) is consistent with previously reported values for devices with IEM of similar thickness ^511^. Devices with thicker ion exchange membranes can achieve higher ON/OFF ratio, at the cost of higher ionic resistance and higher operating voltage^26^. We also notice for different *C*_*D*_, the ON/OFF ratio is less dependent on the fixed charge concentration *C*_*IM*_. This is due to the fact that with higher *C*_*IM*_, more sites in the IEM are available to attract drug ions. As a result, the increased electrostatic attractions between the fixed charge polymers on the IEM and drug ions would increase the amount of drug transported for both active pumping and passive diffusion (amount of transported drugs at steady-state between ON and OFF states can be found in Fig. S1), and the ON/OFF ratio is therefore much less dependent on *C*_*IM*_ compared to *C*_*D*_.

## Device optimization

Based on the contour plots for each of the performance indices presented in this paper, the electrophoretic drug delivery device can be optimized for different disease models based on different desired device characteristics. There isn’t a way to maximize all three performance indices at once. The amount of transported drug *Q* can be increased by increasing *C*_*D*_ and *C*_*IM*_, but due to capacitive charging of the electrodes, *Q* is predominantly limited by the active pumping time, and the effect of increasing *C*_*D*_ and *C*_*IM*_ on transporting more drug is limited. The pumping efficiency *η* is determined by the concentration ratio between *C*_*D*_ and *C*_*IM*_, and the device can reach a high pumping efficiency once the lower concentration ratio limit of 10:1 between *C*_*IM*_ to *C*_*D*_ is reached. The ON/OFF ratio is highly dependent on *C*_*D*_ and much less so on *C*_*IM*_. Devices with higher *C*_*D*_ lead to higher amount of passive leakage, which is the dominant factor for a device to have a lower ON/OFF ratio at higher initial drug concentration.

In the context of tailoring the device performance based on different diseases, applications such as delivering chemotherapy for cancer treatment may require a large dosage in a short period of time. Accordingly, the results presented here indicate such devices should be loaded with a high initial drug concentration *C*_*D*_ to maximize *Q*. Also, considering the short active pumping time due to capacitive charging, it is preferable for the device to have a high pumping efficiency *η* by having higher *C*_*IM*_, so that the applied potential is pumping predominately the drug. In contrast, for chronic treatments such as seizure control for which minimal passive leakage may be a requisite for safe and long-term implantation, a lower initial drug concentration *C*_*D*_ is desirable to achieve a high ON/OFF ratio for electrophoretic drug delivery devices.

## Discussion

Much advancement for electrophoretic drug delivery devices relies on membrane materials development and concentration ratio optimization between the drug and the fixed charge groups in the IEM. The capacitive charging in the electrodes and selectivity of IEM dictates the devices characteristics for electrophoretic drug delivery devices.

One might think that having higher initial drug concentration *C*_*D*_ in the electrophoretic drug delivery device is always advantageous, since devices with higher *C*_*D*_ can obtain higher instantaneous drug delivery. Also, the lifetime of the device can be prolonged by having higher *C*_*D*_ in the source reservoir. However, having higher initial drug concentration *C*_*D*_ in the device would lead to worse pumping efficiency *η* and worse ON/OFF ratio which may not be preferable for chronic treatments. Likewise, even though having higher fixed charge concentration *C*_*IM*_ in the IEM can increase the pumping efficiency *η* and the amount of transported drug *Q* when actively pumping, it can also lead to drawback such as higher leakage when device is switched off.

Looking ahead, we see that the device parameters in terms of concentration for current electrophoretic drug delivery devices sit in the middle part of the contour plots. (Most commercially available ion exchange membrane materials typically have a fixed charge concentration in the range between 100-1000 mM^21^, whereas drug concentration commonly used in electrophoretic drug delivery devices ranges from 10-100 mM^5,22^). One can increase the device performance by either pushing for higher fixed charge concentration, higher drug solubility, or higher concentration ratio between drug and ion exchange membrane with lower initial drug concentration.

## Conclusion

We present a one-dimensional computational model to illustrate the underlying physics and optimal materials parameters for electrophoretic drug delivery devices incorporating an ion exchange membrane. The capacitive nature of the electrodes is incorporated in the model thereby allowing for a depiction of the temporal dependence of drug delivery under experimentally relevant conditions. The results indicate that the device can be optimized for maximum drug transported by having high *C*_*D*_ and high *C*_*IM*_. For chronic treatment, the device should be optimized for high ON/OFF ratio and high pumping efficiency by having lower initial drug concentration and high fixed charge concentration for the ion exchange membrane to be selective and prevent passive leakage of drugs when the device is switched off. Finally, we suggest future improvements for electrophoretic drug delivery devices and materials development for ion exchange membranes used in the context of different diseases.

## Supplementary information

Supplementary information.
