# Supplementary material for: Materials and Device Considerations in Electrophoretic Drug Delivery Devices
Source: Sci Rep. 2020 Apr 28;10:7185. doi: 10.1038/s41598-020-64114-0 (PMC7188809; doi:10.1038/s41598-020-64114-0)
Supplement: Supplementary file 1 — Supplementary information. [file 41598_2020_64114_MOESM1_ESM.docx]

Supporting Information

Materials and Device Considerations in Electrophoretic Drug Delivery Devices

Shao-Tuan Chen, Christopher M. Proctor*, George G. Malliaras*

The governing equations of the computational model is described as below:

Let subscript *i* represent the *i^th^* ion in the system. The general form of the Nernst-Planck equation describing the concentration gradient of the *i^th^* species is written as:

 (1)

where *c* is the concentration, *t* represents time and *j* is the magnitude of the ionic flux.

The ionic flux *j_i_* subjected to diffusion and electromigration can be also written as:

 , (2)

where *D* represents the ionic diffusivity, *z* represents the valence of the ion, *e* is the elementary charge, k_B_ is the Boltzmann constant, *T* represents temperature, and φ is the voltage.

The Poisson’s equation relates the charge density *ρ* to the resulting electric field *E* by:

, (3)

where *ε* is the dielectric constant, and relationship between electric field E and potential φ is given by:

. (4)

Equations 1 to 4 constitute the governing equations for the one-dimensional model in this study.

**Table S1.** List of variables used in this study.

**Figure S1.** Amount of drug transported at steady-state for different *C_IM_* when the device is ON (left) and OFF (right).
